# Role of scaling combination of risk factors in clinical and imaging findings during pregnancy in predicting placenta accreta spectrum

**DOI:** 10.22088/cjim.13.1.10

**Published:** 2022

**Authors:** Afsaneh Mohammadi, Zinatossadat Bouzari, Karimollah Hajian-Tilaki, Mehrdad Nabahati, Rahele Mehraeen

**Affiliations:** 1 Department of Radiology, Babol University of Medical Sciences, Babol, Iran; 2 Infertility and Reproductive Health Research Center, Health Research Institute, Babol University of Medical Sciences, Babol, Iran; 3 Social Determinants of Health Research Center, Health Research Institute, Babol University of Medical Sciences, Babol, Iran; 4 Non-Communicable Pediatric Disease Research Center, Health Research Institute, Babol University of Medical Sciences, Babol, Iran

**Keywords:** Placenta accreta, Clinical findings, Imaging findings, Ultrasound, Magnetic resonance imaging, pregnant women, Prediction, Combination

## Abstract

**Background:**

Placenta accreta is one of the known causes of maternal mortality and morbidity. If diagnosed before delivery, appropriate actions can be taken. The aim of this study was to investigate the role of scaling combination of risk factors in predicting placenta accreta spectrum (PAS).

**Methods:**

In this cross-sectional study, 120 pregnant women with two criteria and more of placenta previa in their ultrasound, underwent MRI. Clinical scores (history of surgery, cesarean section, previa, etc.) and paraclinical scores (ultrasound and MRI) were recorded and combined. In cases of hysterectomy, pathological examination was performed. The results were compared and analyzed using SPSS Version 22. The significance level was less than 0.05.

**Results:**

Of the120 studied patients, 90 (75%) women were diagnosed with placenta previa in which, 32(36%) patients had placenta accreta and 12 patients had placenta accreta without placenta previa. The mean ultrasound score in women without and with placenta accreta were 0.05±0.32 and 2.43±1.83 (p<0.001). The mean MRI score in women without and with placenta accreta were 0.05±0.27 and 2.07±2.02, respectively. The cut-off point, sensitivity and specificity were 0.50, 100% and 93.4%, respectively. The mean clinical score without and with placenta accreta were 1.97±1.32 and 4.89±3.21, respectively. The cut-off point, sensitivity and specificity were 2.50, 70% and 80%, respectively. The cut-off point of combination score, sensitivity and specificity were 3.50, 89%, 83%.

**Conclusion:**

The results of the present study showed that the most specific test to confirm the definitive diagnosis of placenta accreta is paraclinical score, alone.

Placenta accreta is a known cause of maternal mortality and morbidity (1) and one of the two causes of postpartum hemorrhage and the most common indication for hysterectomy (2, 3). Placenta accreta spectrum (PAS) is a general term used to describe placentaaccreta, increta, and percreta; including attachment of the placenta to myometrium without intervening decidua (accreta), the invasion of the myometrium (increta), and the infiltration of the surrounding organs through the uterine serosa (percreta). The exact etiology of placenta accreta is unknown, however, cesarean section and placenta previa have been cited as the most important risk factors (4-9). In the absence of placenta previa, although the likelihood of PAS following a previous cesarean section is lower, the rate of PAS increases with increase in multiple repeat cesarean sections (9).

Other risk factors for PAS include history of uterine surgery (myomectomy that entered the uterine cavity, hysteroscopic resection to remove intrauterine adhesions, cornual resection for ectopic pregnancy, dilation and curettage procedure, and endometrial ablation), cesarean scar pregnancy (CSP), maternal age over 35, manual removal of the placenta, postpartum endometritis, infertility, and infertility treatments (7, 10-11). Due to the abnormal adhesion of the placenta to the myometrium in placenta accreta,there has been an increased risk of severe bleeding when the placenta comes out, which requires blood transfusion and hysterectomy to save the mother’s life (2). The placenta accreta has an overall prevalence of 0.04 and may not even be definitively diagnosed until hysterotomy is done (8).

For diagnosis during pregnancy, some studies have suggested sensitivity of placental lacunae, multiple levels of hypoechogenicity, and abnormal size of placenta in ultrasound (12, 13). MRI also reveals the criteria for the detection of accreta (12) including the presence of intraplacentalbands on T2 weighted images, abnormal uterine bulging, focal interruption of the myometrium and infiltration pelvic organs.

Given that the presence of placenta accreta places the pregnancy in the category of high-risk pregnancies, and the fact that there has been no consensus on the treatment of this problem, the best way to deal with this problem is predicting placenta accreta during pregnancy. Considering the importance of diagnosing PAS before delivery and to adopt the best treatment method to reduce maternal mortality and morbidity, the present study was conducted to combine the scaling of risk factors in clinical findings, ultrasound signs and MRI findings and to determine the appropriate cut-off point based on prenatal score in predicting placenta accreta.

## Methods

This cross-sectional study was approved by the Ethics Committee of Babol University of Medical Sciences with the code IR.MUBABOL.HRI.REC.1398.200 and was conducted among the 120 pregnant women with a diagnosis of placenta accreta that referred to Ayatollah Rouhani Medical Center. Pregnant women in their first trimester with an initial ultrasound based on previa or a history of uterine manipulation, repeat cesarean section, and women with confirmed placentation in their ultrasound performed at the same center were included in the study and examined until the infant was born. The women with no access to complete delivery data were excluded from the study. Abdominal ultrasound was performed at 18-22 weeks of gestational age. If the placenta was low-lying, another ultrasound was performed at 28 weeks to confirm previa and if it was positive, women were asked to participate in the study. 

The GE Voluson 730 Ultrasound System was used based on the combination of 3.5 MHz convex abdominal probe and 7.5 MHz vaginal probe through abdominal and vaginal scanning combined with color Doppler ultrasound to evaluate placenta. Each patient with two criteria (5, 6) and more was placed in the high probability category of placenta accreta and needed further imaging, they underwent MRI without contrast by Signa HDxT 1.5 Tesla (General Electric Healthcare). The sequences in axial, sagittal and coronal planes were taken relative to the studied organ. Images T1 and T2 were also taken (14). MRI images were examined by a radiologist with 10 years of experience in MRI of placenta. After observing signs of placenta accreta in MRI images (15, 16), the case was reported as positive. 

In cases resulting in hysterectomy, the sample was thoroughly examined in terms of pathology. All information including maternal age, smoking and alcohol use, medical history, maternal height and weight, gestational age, number of deliveries, number of children, number of abortions, history of placental abruption, time of diagnosis, history of uterine surgery, findings and outcomes during surgery and after that, as well as ultrasound and MRI findings were recorded in the patient’s questionnaire. The clinical and paraclinical scale is scored to predict PAS and the scores are summed up: Data were analyzed using SPSS V.22, Chi-square, Mann-Whitney and ROC Curve statistical tests. CAT maker software was also used to determine the diagnostic value and p<0.05 was considered significant. 

## Results

The mean age of patients was 33.19±2.79 years (minimum age of 28 and maximum age of 38 years). Of the 120 studied patients, 90 (75%) women were diagnosed with placenta previa. Among the 90 patients diagnosed with placenta previa, 32 (36%) patients had placenta accreta and 12 patients had placenta accreta without placenta previa.

The samples were examined after cesarean section according to pathology, which is the diagnostic gold standard for placenta accreta, and a total of 44 (36.6%) cases of placenta accreta were diagnosed, of which 32 cases were placenta previa. Among the 44 patients with a diagnosis of placenta accreta, the placenta was anterior in 25 (56.8%) cases and posterior in 19 (43.2%) cases. None of the studied patients were smokers or drank alcohol. Placental abruption was also not reported in any of the cases. All but 3 (2.5%) had a history of cesarean section and other uterine surgeries and all patients underwent MRI and finally surgery.

In the study of ultrasound indices, except for disruption of the bladder line, there was a significant difference between women with placenta accreta and women without placenta accreta in other cases (table 1). 

**Table 1:** Comparison of ultrasound criteria in patients based on diagnosis of placenta accrete

P value*	**Placenta Accreta**		**Total** **N(%)**	**Variable**
	**Yes** **N(%)**	**No** **N(%)**		
				Loss of the clear zone
<0.001	20(45.5) 24(54.5)	76(100) -	96(80) 24(20)	No Yes
				Myometrial thinning
<0.001	22(50) 22(50)	- 76(100)	98(81.7) 22(18.3)	No Yes
				Abnormal Vascularity
<0.001	17(38.6) 27(61.4)	- 76(100)	93(77.5) 27(22.5)	No Yes
				Placental Bulge
<0.001	36(81.8) 8(18.2)	76(100) -	112(93.3) 8(6.7)	No Yes
				Disruption of bladder line
0.62	42(95.5) 2(4.5)	74(97.4) 2(2.6)	116(96.7) 4(3.3)	No Yes
				Placental Lacunae/Exophytic mass
<0.001	33(75) 11(25)	76(100) -	109(90.8) 11(9.2)	No Yes

*chi – square test

The mean score of ultrasound criteria in women without placenta accreta diagnosis was 0.05±0.32 and in women with placenta accreta diagnosis was 2.43±1.83, which was significantly higher in women with placenta accreta diagnosis (p<0.001). In the study of MRI indices, except for abnormal placental vascularity and infiltration of the pelvic organ, there was a significant difference between women with and without diagnosis of placenta accreta in other cases (table 2).

**Table 2:** Comparison of MRI criteria in patients based on diagnosis of placenta accrete

**Variable**	**Total** **N(%)**	**Placenta Accreta**		**P value** ^*^
		**No** **N (%)**	**Yes** **N(%)**	
Abnormal uterine bulging No Yes	104(86.7) 16(13.3)	76(100) -	28(63.6) 16(36.4)	0.02
Heterogenous signal intensity within the placenta				
No Yes	84(70) 36(30)	76(100) -	8(18.2) 36(81.8)	<0.001
Abnormal placentalvascularity				
No Yes	119(99.2) 1(0.8)	75(98.7) 1(1.3)	4(100) -	0.44
Focal interruption of themyometrium				
No Yes	112(93.3) 8(6.7)	75(98.7) 1(1.3)	37(84.1) 7(15.9)	0.002
Presence of intraplacentalbands on the T2-W imaging				
No Yes	106(88.3) 14(11.7)	75(98.9) 1(1.3)	31(70.5) 13(29.5)	<0.001
Infiltration pelvic organ				
No Yes	118(98.3) 2(1.7)	76(100) -	42(95.5) 2(4.5)	0.06

*chi – square test

**Figure1:**
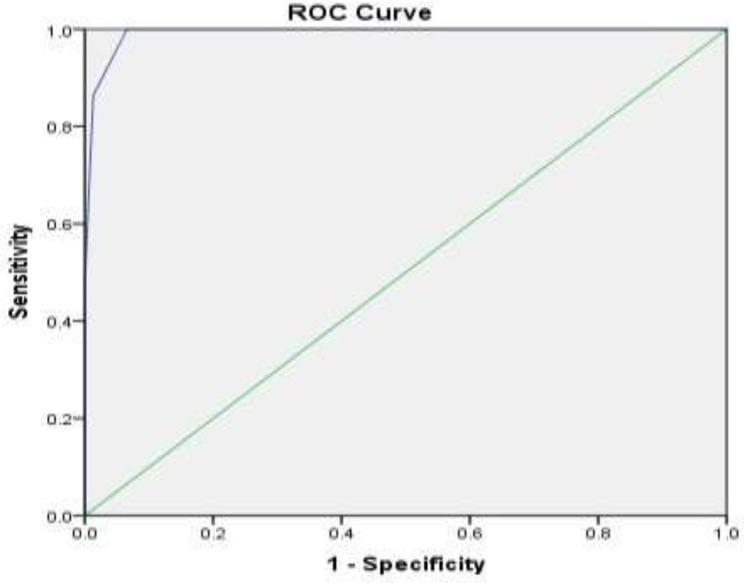
The ROC curve shows the relationship between specificity and sensitivity of paraclinical score in placenta accreta diagnosis

The mean MRI score in women without placenta accreta was 0.05±0.27 and in women with placenta accreta was 2.07±2.02, which was significantly higher in women with placenta accreta (p<0.001). The mean paraclinical score in women without placenta accreta was 0.11±0.41 and in women with placenta accreta was 3.50±3.77, which was significantly higher in women with placenta accreta (p<0.001). Based on the evaluation of the ROC curve diagram, the cut-off point for the paraclinical score was calculated to be 0.50, based on which a sensitivity of 100% and a specificity of 93.4% were obtained (figure 1) (AUC = 0.99, CI: 0.98-1, P=0.001).

The sensitivity of the paraclinical score in predicting placenta accreta is 100% and its specificity is 93%. It should be noted that all women had ≥ 2 parity. Furthermore, none of the women were smokers and did not have postpartum endometritis, infertility, various uterine pathologies and a history of cesarean section more than 3 times. In the study of clinical score indices between women with and without placenta accreta, except for age over 35 years, blood pressure disorders and cesarean scar pregnancy were observed in other cases (table 3).

The mean clinical score in women without placenta accreta was 1.97±1.32 and in women with placenta accreta was 4.89±3.21, which was significantly higher in women with placenta accreta diagnosis (p<0.001). 

**Figure 2:**
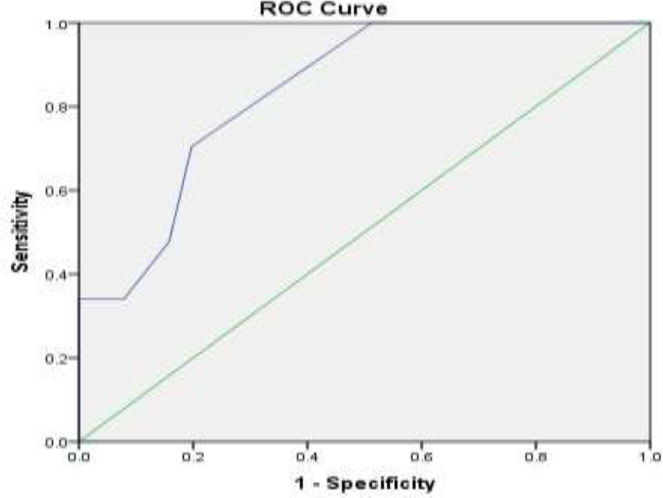
The ROC curve shows the relationship between clinical score specificity and sensitivity in placenta accreta diagnosis

Based on the evaluation of the ROC curve diagram, the cut-off point for the clinical score was calculated to be 2.50, based on which a sensitivity of 70% and a specificity of 80% were obtained (figure 2) (AUC=0.83, CI: 0.77-0.90, P=0.001). 

The sensitivity of the clinical score in predicting placenta accreta is 70% and its specificity is 80%. The mean overall score (scaling combination) in women without placenta accreta diagnosis was 2.08±1.36 and in women with placenta accreta diagnosis was 9.39±6.86, which was significantly higher in women with placenta accreta diagnosis (p<0.001).

**Table 3:** Comparison of clinical criteria in patients based on diagnosis of placenta accrete

P value^*^	Placenta Accreta		Total N(%)	Variable
	**Yes** N(%)	**No** N(%)		
				Age>35
0.40	30(68.2) 14(31.8)	46(60.5) 30(39.5)	76(63.3) 44(36.7)	No Yes
				Blood pressure of Disorders
0.82	41(93.2) 3(6.8)	70(92.1) 6(7.9)	111(92.5) 9(7.5)	No Yes
				Manual removal of the placenta
<0.001	33(0.75) 11(0.25)	76(100) -	109(90.8) 11(9.2)	No Yes
				Baby Girl
<0.001	23(52.3) 21(47.7)	76(100) -	99(82.5) 21(17.5)	No Yes
				History Of 1 C/S
<0.001	18.40.9) 26(59.1)	76(100) -	94(78.3) 26(21.7)	No Yes
				History Of 3 C/S
<0.001	27(61.4) 17(38.6)	76(100) -	103(85.8) 17(14.2)	No Yes
				History Of Uterine Surgery
<0.001	29(65.9) 15(34.1)	76(100) -	105(87.5) 15(12.5)	NO Yes
				Pregnancy in C/S
0.69	43(97.7) 1(2.3)	75(98.7) 1(1.3)	118(98.3) 2(1.7)	No Yes

*chi – square test

**Figure 3:**
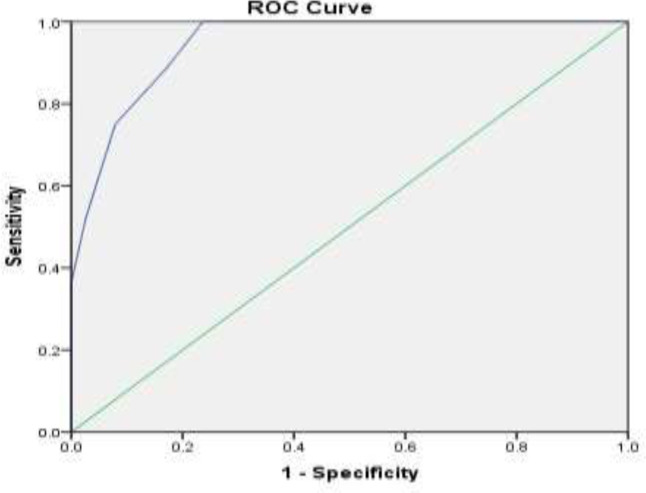
The ROC curve shows the relationship between specifity and overall score sensivity in placenta accreta diagnosis

Based on the evaluation of the ROC curve diagram, the cut-off point for the overall score was calculated to be 3.50, based on which a sensitivity of 89% and a specificity of 83% was obtained (figure 3) (AUC=0.94, CI: 0.91-0.98, P=0.001).

The sensitivity of the overall score in the prediction of placenta accreta is 89% and its specificity is 83% (table 4).

**Table 4:** Diagnostic value of the overall score in placenta accreta prediction

Accuracy	LR- CI 95%	LR+ CI 95%	NPV CI 95%	PPV CI 95%	Specificity CI 95%	Sensivity CI 95%	Variable
% 85	0/14 0/06-0/31	5.18 3.12-8.60	% 93 86-99	% 75 63-87	% 83 74-91	% 89 79-98	Total Score

## Discussion

The prevalence of placenta accreta in the present study was 36.7% in 120 samples. In the study of Shawky et al., the prevalence of placenta accreta among 50 cases was 36% (17), similar to the present study. Ayati et al. of 82 cases, identified 21% placenta accreta (18), lower than the present study, which may be due to differences in diagnostic methods. 

Fitzpatrick et al. reported the history of placenta previa, history of uterine surgery, blood pressure disorders, smoking, and female infant, as the clinical risk factors for placenta accreta (7). While in the present study, curettage suction, manual removal of the placenta were also identified as risk factors. Our results showed that age over 35 years, hypertension and cesarean scar pregnancy are not the risk factors for placenta accreta, while Knight et al. introduced advanced maternal age as a risk factor (19), which may be due to the fact that the mean age in Knight’s study was higher than the present study. 

In this study, since the diagnosis accuracy of clinical score is 76%, it is better to use it to diagnose placenta accreta in combination with imaging findings. The weak point of the present study was that in the evaluation of ultrasound indices, disruption of bladder line was not seen as risk factors for placenta accreta. However, the frequency of this factor is lower than other criteria and can be one of the reasons for the insignificance of this factor. The mean difference of ultrasound score in women with and without placenta accreta diagnosis is very large (2.39 to 0.03) and therefore ultrasound is one of the most useful diagnostic methods in this field. 

In the study of Ayati et al., the sensitivity of Doppler ultrasound and MRI were 87% and 76%, respectively, and their specificity were 63% and 83%, respectively. They concluded that women with high risk factors for placenta accreta should undergo Doppler ultrasound as a first step. (18). The mean difference in MRI scores was also significant between women with and without placenta accreta (2.07 to 0.05), which is why MRI is used as gold standard for placenta accreta diagnosis (20, 21). Abnormal placental vascularity and pelvic organ infiltration were not recognized as risk factors in MRI scores due to the low number of cases of these factors. Then, by summing up ultrasound and MRI scores, the paraclinical score was examined as the main scale for placenta accreta diagnosis. 100% sensitivity and 93.4% specificity of paraclinical score at the cut-off point of 0.5 and 95.83% accuracy indicate the reliability of paraclinical score. Regarding negative predictive value of 93%, this index helps us to use this factor for triage of patients with placenta accreta. The diagnostic accuracy of the overall score is 85%, which is higher than the accuracy of the clinical score alone but lower than the paraclinical score. 

The results of the present study showed that the most specific test to confirm the definitive diagnosis of placenta accreta is the paraclinical score. A study with a larger sample size and higher frequency of each of the clinical and paraclinical diagnostic criteria is recommended. 

## Funding:

This study was financially supported by Babol University of Medical Sciences.

## Conflicts of interest:

The authors declare that there is no conflict of interest.
